# The Huxley Lecture of Recent Advances in Science and Their Bearing on Medicine and Surgery*Delivered at the opening of the Charing Cross Hospital Medical School on October 3

**Published:** 1898-11

**Authors:** Rudolf Virchow

**Affiliations:** University of Berlin


### Selections and Abstracts.

### THE HUXLEY LECTURE OF RECENT ADVANCES IN SCIENCE AND THEIR BEARING ON MEDICINE AND SURGERY.*

By Prof. Dr. RUDOLF VIRCHOW, University of Berlin.

The honor of being invited to deliver the second Huxley Lecture has deeply moved me. How beautiful are these days of remembrance which have become a national custom of the English people, and through which the memory of intellectual heroes is kept alive to posterity! How touching is this act of gratitude when the celebration is held at the very place wherein the genius of the man whom it commemorates was first guided towards its scientific development! We are filled not alone with admiration for the hero, but at the same time with grateful recognition of the institution which planted the seed of high achievement in the soul of the youthful student.

That you, gentlemen, should have entrusted to a stranger the task of giving these feelings expression seemed to me an act of such kindly sentiment implying such a perfect confidence that I at first hesitated to accept it. How am I to find in a strange tongue words which shall perfectly express my feelings? How shall I in the presence of a circle of men who are personally unknown to me, but of whom many knew him who has passed away, and had seen him at work, always find the right expression for that which I wish to say as well as a member of that circle itself could? I dare not believe that I shall throughout succeed in this. But if in spite of all I repress my scruples it is because I know how indulgently my English colleagues will judge my often incomplete statements, and how fully they are inclined to pardon deficiency in diction, if they are convinced of the good intentions of the lecturer. I may assume that such a task would not have been allotted to me had not those who imposed it known how deeply the feeling of admiration for Huxley is rooted within me, had they not seen how fully I recognized the achievements of the dead master from his first epoch-making publications, and how greatly I prized the personal friendship which he extended towards me. In truth, the lessons that I received from him in his laboratory—a very modest one according to present conditions—and the introduction to his work which I owe to him, form one of the pleasantest and most lasting recollections of my visit to Kensington.

---

*Delivered at the opening of the Charing Cross Hospital Medical School on October 3

The most competent witness of Huxley's earliest period of development, Professor Foster, presented in the first of these lectures a picture of the rapidly increasing extension of the biological knowledge, which must have excited not only our admiration but also the emulation of all who study medicine. Upon me the duty is incumbent of incorporating with this presentment the newer strides of knowledge, and of stating their influence upon the art of healing. So great a task is this that it would be presumptuous even to dare to attempt its accomplishment in a single lecture. I have decided, therefore, that I must confine myself to merely sketching the influence of biological discoveries upon medicine. In this way also will the example of Huxley be most intelligible to us.

Huxley himself, though trained in the practical school of Charing Cross Hospital, won his special title to fame in the domain of biology. As a matter of fact, at that time even the name of biology had not come into general use. It was only recently, as I showed in my lecture "On the Position of Pathology amongst Biological Studies,"* that the idea of life itself obtained its full significance. Even in the late middle ages it had not sufficient strength to struggle through the veil of dogmatism into the light. I am glad to be able to-day, for the second time, to credit the English nation with the service of having made the first attempts to define the nature and character of life. It was, as I then pointed out, Francis Glisson, who, following expressly in the footsteps of Paracelsus, investigated the principium vitæ. If he could not elucidate the nature of life he at least recognized its main characteristic. This is what he was the first to describe as "irritability," the property on which the energy of living matter depends. He thus succeeded in setting aside the mystical idea of the spiritualistic Archæus which Paracelsus and his followers had placed in the forefront of their deliberations, and in locating the principium energeticum in matter itself.

How great was the step from Paracelsus to Glisson and—we may continue—from Glisson to Hunte·! According to Paracelsus, life was the work of a special spiritus which set material substance in action, like a machine; for Glisson matter itself was the principium energeticum. Unfortunately, he did not confine this dictum to living substances only, but applied it to substance in general, to all matter. It was Hunter who first announced the specific nature of living matter as contrasted with non-living. But he also did not attain perfect clearness of vision, because in the development of English medicine the idea had been allowed to take root and grow, that life was not bound up with structure, so that Hunter also was led to place a materia vitæ diffusa at the head of his physiological and pathological views. Hence he arrived at

*Croonian Lecture, *Proc. Roy. Soc.* Vol. LIII.

the assumption of the so-called plastic substances in respect of which the blood served both as rallying-point and seat of formation, and so it happened that in place of the old Greek humoral pathology which Paracelsus had overthrown, a new humoral pathology arose—hematology. According to the teaching of Hewson and Hunter, the blood supplied the plastic materials of physiology as well as the plastic exudatas of pathology.

Such was the basis of the new biological method, if one can apply such an expression to a still incomplete doctrine, in 1842 when Huxley was beginning his medical studies at Charing Cross Hospital. Of pathology in England, John Hunter was the accepted master, and so remained for many years thereafter. So great was his influence that Continental medicine also recognized it. To this I can myself testify, as I was at this time at the end of my university studies. It would lead too far afield were I to recount in this place how it happened that I myself, like Huxley, was early weaned form the pernicious doctrines of humoral pathology; it must suffice to say that salvation lay in the same science which had already once before, in the sixteenth century, brought about deliverance from humoral pathology. It then came about that Vesalius threw over the authority of Galen and founded human anatomy upon direct observation, on necropsy. Since then anatomical studies have been much widened and improved. When Huxley himself left Charing Cross Hospital, in 1846, he had enjoyed a rich measure of instruction in anatomy and physiology. How great this was can be gathered from the interesting statistics which Professor Foster has collected with the aid of Huxley's distinguished fellow student, Sir Joseph Fayrer. Of the lectures which junior students attended, 140 in each of the three years of study were devoted to anatomy and physiology. Thus trained, Huxley took the post of naval surgeon, and by the time that he returned, four years later, had become a perfect zoologist and a keen-sighted ethnologist. How this was possible any one will readly understand who knows from his own experience how great the value of personal observation is for the development of independent and unprejudiced thought. For a young man who, besides collecting a rich treasure of positive knowledge, has practiced dissection and the exercise of a critical judgment, a long sea-voyage and a peaceful sojourn among entirely new surroundings afford an invaluable opportunity for original work and deep reflection. Freed from the formalism of the schools, thrown upon the use of his own intellect, compelled to test each single object as regards properties and history, he soon forgets the dogmas of the prevailing system and becomes first a sceptic and then an investigator. This change, which did not fail to affect Huxley, and through which arose that Huxley whom we commemorate to-day, is no unknown occurrence to one who is acquainted with the history not only of knowledge but also of individual scholars. We need only point to John Hunter and Darwin as closely allied examples.

The path on which these men have achieved their triumphs is that which biology in general has trodden with ever-widening strides since the end of last century; it is the path of genetic investigation. We Germans point with pride to our countryman who opened up this road with full conviction of its importance, and who directed towards it the eyes of the world—our poet-prince Goethe. What he accomplished in particular for plants others of our fellow-countrymen achieved for animals. I recall Casper Friedrich Wolf, Dolinger, Joh. Friedrich Meckel, Carl von Baer, and our whole embryological school. As Harvey, Haller and Hunter had once done, so these men began also with the study of the "ovulum," but this very soon showed that the egg was itself organized, and from it arose the whole series of organic developments. When Huxley after his return came to publish his fundamental observations, he found the history of the progressive transformations of the contents of the egg already verified, for it was by now known that the egg was a cell, and that from it fresh cells, and from them organs, arose. The second of his three famous papers, that on the relationship between man and the animals next beneath him, limned in exemplary fashion the parallelism in the earliest development of all animal beings. But beyond this it stepped boldly across the border-line which tradition and dogma had drawn between man and beast. Huxley had no hesitation in filling the gap which Darwin had left in his argument, and in explaining "that in respect of substance and structure man and the lower animals are one."

Whatever opinion one may hold as to the origin of mankind, the conviction as to the fundamental correspondence of human organization with that of animals is at present universally accepted. All biological science, especially physiology and pathology, creates hence the impulse to corresponding studies, and in particular all that has to be based on experiment must in the first instance be investigated in animals, while all that requires morphological confirmation finds support in comparative anatomy, histology and embryology. The basis of our comprehension of the theory of medicine actually rests nowadays on minute microscopy, for the elaboration of which the animal tissues form an indispensable control-object. Suffice it to say that in scientific biology the division between man and beast becomes less and less defined, but only let it be remarked the division between the abstract man and the abstract animal. It is the same relation as meets us in the differentiation between plants and animals. How many definitions of this have been put forth in the course of time, and how one after the other has been wrecked! But if we place a given animal and a given plant side by side we overcome the difficulties which we had only raised by our own definition.

The greatest difficulty in biology has arisen in this way—that mankind, following a natural tendency, has set the search after the unitary basis of life in the foreground of its consideration. As a matter of fact, what is more natural than the conclusion that life as a special phenomenon must also have a special basis, and that the material process of life must be derived from a common cause? During the last century an attempt was made to satisfy this claim by the assumption, with ever-increasing conviction, of a special force—vital force. Nowadays we can still perceive the logical errors which this assumption rendered possible. Time has, however, passed judgment upon it, and to-day no one continues to speak of vital force. And yet the necessity for a single basis of all vital manifestations remains. How is this to be satisfied? This is a question which is not alone of great theoretical interest, but which has become an indispensable foundation for practical work, and particularly for medical practice. But in order to reach this foundation, it is first of all necessary to dispense with all the dogmas of the schools, and to seek to construct an objective picture of the nature of vital processes.

As regards material construction, man and the higher animals and plants are no unitary, simple beings; on the contrary, they are put together from many units. They are hence called organisms. If they possess but one single power which set all their parts in action, it would be impossible to understand how the special kind of activity which each one of these organisms exercises in its individual way comes about. This specific activity is present in the organism not alone in its perfect or fully grown form, but also during its development and growth. How can a single power, whether we call it in the spiritualistic sense spirit, soul, spiritus rector, or, in the physical sense, vital force or electricity, build up such diverse organisms? Or if this power resided in a single organ—were it in brain, or spinal cord, or heart—how could those brainless and heartless creatures be explained which present so abnormal a condition that at the beginning of this century they were the proper battleground of the mystics?

There is here, in my opinion, only one solution possible. The life possessed by the higher organisms is not a single one. Their life and all their activities only become intelligible when we go back to the exact representation, based upon a kind of instinctive observation, of the life of their parts. Each constituent part of a living organism has its special life, its vita propria. No one of the older authors proclaimed this more distinctly than Paracelsus. But he at once undid this good idea again by attributing to each living part a particular spiritus, a special Archæus. The best of the succeeding biologists were also held by this notion as in a snare; instead of busying themselves in the observation of vita propria—that is, the action of the parts—they continued to devote themselves to research on the Archæus.

The advances in general science based upon personal observation, and particularly those in medicine, have completely turned the attention of true observers to the study of individual parts. As I pointed out at the Medical Congress at Rome,* it stands most clearly revealed in the history of pathology that the division of the body first into larger regions (head, breast, abdomen, etc.), then into organs, then into tissues, and finally into cells and cell-territories, was the first step which opened up to us the comprehension of disease. The study of regions was followed by that of organs, and this again by that of the tissues, and finally by the cellular theory. But what is true of pathology must hold also for physiology, and as a matter of fact physiology has passed through the same developmental phases, One gradually comes to understand that the life of the individual parts is perfectly clear if one looks away from the Archæi of the organs, or the tissues, and keeps in view only the life and activities of the single cells. For the life of an organ is naught else than the sum of the lives of the single cells which are gathered together into it, and the life of the whole organism is not an individual but a collective function.

If such a collective being is analyzed, no matter whether it be the whole organism, or a single organ, or only one tissue which is presented in its vital activity, the first requisite for a correct interpretation is that one should discard the fabled unity, and should regard the single parts, the cells, as the factors of existence. Single cells can be separated out even in a complex organism, but we should with difficulty arrive at a satisfactory theory if we did not also meet with single free-living cells in Nature. These have provided the first basis for objective investigation. Uni-cellular plants and animals have during this century been continnally more fully and better studied. Botanists and zoologists have become the teachers of physiologists and pathologists. The ova of animals and the corresponding germ-cells of plants have bridged the gap between isolated living cells and higher organisms. It was the recognition of this fact which first raised the famous theorem of Harvey to the high position which it merits.

In a medical school, where the teaching is almost entirely concerned with human beings, we might put this sentence at

*Morgagni and Anatomical Thought.

the head of the lesson: "The organism is not an individual but a social mechanism." An exact anatomical analysis of this mechanism always brings us at last to cells; they are the ultimate constituents of all tissues as they were their origins. Hence we call them the living elements, and hence we regard them as the anatomical basis of all biological analysis, whether it has a physiological or a pathogical object in view.

In relation to this two propositions must be stated: (1) That every organism, like every organ and tissue, provided it is alive, contains cells; (2) that the cells are composed of organic chemical substances, which are not themselves alive, but the mechanical arrangement of which determines the direction and power of their activity.

The first proposition has of late slowly come to be realized. Schwann, who recognized the origin of tissues from cells, still clung to the opinion that in the further development of many tissues the cells were used up. Among these he reckoned that important group which has subsequently become known as the supporting tissues, because it ensures form and stability to single organs, and to the whole organism. First among these stand the osseous and connective tissues, which also form so large a fraction in the quantitative constitution of higher organisms. The conception of the osseous and connective tissues as free from cells must now be given up. Where formerly only empty spaces or mere leaks (lacunæ, holes) were seen in the tissue, we now can demonstrate actual cells. We can even isolate them. Hence it is now desirable that the name "tissue," in the sense of living tissue, should be applied only to such parts as contain living cells. Outside the cells the tissue may contain a more or less rich share of organic (chemical) material, but this intercellular or extracellular substance must be regarded as an additional endowment, and not as a vital factor. Such parts as arose originally from living cells, but of which the cells have perished, must be excluded from biological consideration. As examples may be adduced the epidermis and the hair belonging to it, together with the enamel of the teeth. These consist in reality of dead tissue.

As regards the second proposition that no living organic chemical substance exists, the fact has been objected that all living matter is put together from organic chemical materials; but whoever raises this point must have well-nigh overlooked the fact that these two kinds of substances, the living and the non-living, can not be identified with one another. In spite of chemical similarity or even correspondence, they exhibit recognizable differences, not alone physiological, but also mechanical and physical. Thus since the application of dyes has secured us a glimpse of the variety of the finer mechanical, or if one may say it molecular, arrangements of matter, it has become possible to differentiate living and non-living parts de visu. We are admittedly only on the threshold of these investigations, but the latest researches upon ganglion-cells have shown that even beyond the effects of staining differences between living and no-longer living parts may become optically recognizable.

The enthusiasm with which for centuries the doctrine of formative principles and nutritive materials was built up has already become much abated, and has, in part, been entirely abandoned, through the knowledge that no single chemical substance, no kind of nutritive or formative material which can be employed as such, and without further change, for the origination or formation of cells, has ever been found outside the living organism. And yet a chemist of Liebig's importance actually believed that fibrin could be conveyed directly from the meat consumed into the juices of the body, and thence deposited in the tissues. This was a misconception—a relic from the time of the old humoral pathology—which regarded the living body and its constituent parts as arising simply from the coming together of a few ground-substances (humores cardinales). Hence arose the doctrine of plastic materials which were pre-existent in the food and blood. With an obstinacy which was only surpassed by their superficiality, these theorists remained convinced that the plastic materials as such effected the construction and maintenance of living matter. They failed to see that the nutriment taken in had first to be prepared by special juices secreted by the cells of the digestive organs, and that both the digestive material and the plastic substance of the blood were rendered assimilable only by means of a new change, which had to be effected by the agency of the tissue-cells.

The doctrine of plastic material appeared to have gained new strength through Schwann's cell-theory. One must be careful not to misunderstand this designation. Since the cellular theory of animal and plant life has been established, many have maintained that Schwann's cell-theory is identical with it. Not only is this not the case, but the two stand in exact opposition to one another. Schwann assumed, and believed himself to have directly observed the process, that cells arose in undifferentiated matter, in a fluid or a semi-solid mass, in the following way: First, small particles of a firmer kind were separated off, then these came together into little heaps or lumps, by the internal transformation of which a cell-nucleus gradually arose. Round this a new precipitate of firmer substance now slowly accumulated, and from this arose the body of a cell. Hence the original amorphous substance would be the special constructive material, while the nucleus was the true cell-builder; Schwann called the former cytoblastem, the latter cytoblast.

It is obvious that from these premises people must have been logically led to the conclusion that every form of organic tissue or organism, every kind of new cell must be separated from the preceding by a definite gap (hiatus), so that each new formation must be grouped as a discontinuous vital origin. Strangely enough, this classification arose and was accepted at a time when Darwin was already at work proving that new species arise by the modification of pre-existing forms. But Schwann's cell-theory was in truth a resuscitation of the archaic doctrine of spontaneous generation (generatio æquivoca, epigenesis). With the rule of such a creed Darwinism was incompatible.

The supports of this generatio æquivoca have been, as far as zoology is concerned, gradually demolished. The formation of tissue-cells from the egg and its partition has been observed throughout the whole animal kingdom. Apparently eggless animals, such as the cestoids and trichinæ, have one after the other been brought under Harvey's law; we know their eggs, their embryos, and their wanderings. There remains, in fine, but one great domain, though this is of the highest importance; it belongs particularly to pathology, and is that of the plastic exudates, which accompany the most important clinical processes, particularly the inflammatory.

It will readily be understood that so essentially pathological a subject would have but little interest for pure natural philosophers. They left it to medical men, who have to occupy themselves with it all day long. But in medicine this territory was held sacred; no one doubted that therein spoke old, well-attested experience. We old students were endowed with the so-called theorem of the plastic exudates from our earliest studies. Translated into our latter-day parlance, such a theorem would recognize discontinuity in most pathological new formations; it would establish—and this is well worthy of note—the grounds for the dogma of the origin of life from non-living matter. Experience has taught us the exact opposite.

Permit me here, gentlemen, to speak a little more personally than is elsewhere my intention. Perhaps it will be more intelligible to the students of this hospital, and will make more impression if I narrate how I myself arrived at quite other views.

It was towards the end of my academical studies, more than fifty years ago, that I had to take up the work of assistant in the ophthalmic clinic of the Charite Hospital, at Berlin. My attention was at once directed to the diseases of the cornea. We had severe cases of keratitis, but I saw in them no exudation; numerous cataract-operations were performed and the wounds closed, but not by plastic exudation; this was absent from all corneal scars. Could this be explained by the circumstance that the cornea, apart from its circumference, is a non-vascular tissue? My interest was at once focused on the non-vascular tissues. I turned first to the articular cartilages, and behold, here also I found the greatest changes without the presence of exudation, or, at any rate, of plastic exudation. I need only recall the form of inflammation which I named arthritis chronica deformans, and which is described by French physicians as arthrite seche. My experimental studies on the inflammation of the walls of blood-vessels showed that the equally non-vascular intima of the larger arteries, and in part also that of the veins, can undergo great changes without even a trace of exudation being produced. Later on anatomical investigations on endocarditis led to the same result, provided that parietal thrombi were not regarded as exudations. But in all these cases and in every place there were found changes in the tissue-cells, active such as swelling, multiplication of nuclei, etc., or passive as fatty degeneration.

I next turned my attention to vascular organs, and in particular to those which were recognized by pathology as the common seats of exudation-processes. I refer, first, to the medullary infiltration of the lymphatic (follicular) tissues of the intestine and mesenteric glands in typhoid fever so strikingly depicted by the Vienna school; instead of the amorphous albuminons exudate which was described, I found only cells, and cells of the same kind as those which are normally present in these situations. The same was revealed in the so-called caseous exudates which were at one time ascribed to scrofula, at another to tuberculosis; the cheesy material was admittedly in the main amorphous, but it was in reality not an exudation at all, above all, not a primary product of disease, but rather the secondary product of degenerative necrobiotic changes in parts of the tissues, which had formerly been organized, and not infrequently actually hyperplastic.

It is not necessary to go further into details in order to show how great is the realm of this pseudo-exudative process. But I can not help referring to another series of morbid processes affecting the bones. It was whilst studying rickets that I first learnt the biological significance of the cartilage-corpuscles, the nature of which had till then been interpreted in very different ways. I believe that I was the first to distinguish in these corpuscles what must be actually recognized as cells from the merely capsular and extracellular coverings. The rachitic disturbance now brought into fullest evidence an appearance which was repeatedly misunderstood even by later observers; this was the increase of these cells by division, and the consequent growth of the cartilage.

It was not difficult to follow out the direct transition of the epiphysial cartilage into the periostem of the neighboring bone, and thus into connective tissue. At this time the whole world was convinced of the correctness of the statement made by Duhamel that increase in thickness in the long bones was affected by the periosteal vessels exudating a nutritious juice out of which the new bone-substance was formed. Pathologists had extended this formula to periostitis and the formation of exostoses and hyperostoses; they assumed that between the periosteum and the bone a plastic exudation was excreted and stored up, in which the new osteophyte arose by secondary organization. The consequence of my investigations was that in not one of these spots, neither in the cartilage nor in the periosteum, neither in normal growth nor in rickets or periostitis, was organization preceded by the presence of a recognizable amorphous exudation. On the contrary, it was indubitably shown that the first stage of the changes was an active productive process of cell-multiplication; that at the same time the intercellular substance altered in character and underwent a series of successive changes till it assumed an osteoid appearance; and that then, and not till then, followed calcification and true ossification. There was also no difficulty in adducing the proof that the separate stages of these processes in cartilage and in periosteum ran a perfectly parallel course, although the new tissue was in the one case at first true cartilage, in the other only cartilage-like. If one wishes to designate this process in general it must be called proliferation. Most of these processes are of the nature of proliferation. Whoever calls the proliferative layer an exudation will never obtain an objective view of the actual proceeding.

There is thus not the slightest necessity for the genuine observer to hold to the arbitrary and totally erroneous formula of a plastic exudation. There is no such thing as a plastic exudation which is ever simply amorphous; the cells which may be found in it have not arisen there. With this proof, which can be obtained in numberless other places, the doctrine of the discontinuous origin of pathological new formations is set aside. Every such new formation presupposes a tissue from which its cells arise; this is its matrix. There is no difference in principle between the descent of men and animals from one mother and the descent of pathological new formations from one matrix. Pathology has been somewhat late in arriving at the knowledge of this correspondence, but I think that it has acquired especial value for biology in general.

In order to avoid misunderstanding, it may be noted that not every living cell is capable of becoming a matrix. All cells which are destined for the highest animal functions prove sterile, or at least very hypothetically capable of proliferation. Ganglion-cells, primitive muscle bundles, red blood-corpuscles do not come under consideration as regards the theory of pathological descent. The more indifferent cells, on the other hand, above all those of cartilage, connective tissue, and epithelium, exhibit a market proclivity to bring forth new cells. Many cells again, such as bone-corpuscles and fat-cells, require a special preparatory metaplastic stage before they can produce a new brood.

Proliferation is an active property of special cells. That it can not be performed by all cells alike in no way alters the fact that it can only be performed by cells. It is just as little a function of an entire organism, for this itself would then have to be unicellular. In this property lies the explanation of the origin of a whole organism from a single egg-cell, that wonderful process which comes to pass but once in the life of animal. Once tissues have arisen each cell of a matricial tissue may in respect of proliferation be compared to an ovum; it brings forth a new progeny from which new tissue grows. This tissue bears, as a rule, the stamp of its matrix—it is built on the maternal type. This is the nature of descent, and herein lies the key to the knowledge of heredity, that puzzling appearance with which mankind has ever busied itself.

According to the humoral theory heredity was derived from the body-fluids and in particular from the blood. According to this idea the blood furnished the means of the continuance of the family and the race; blood-relationship explained the similarity not only of the juices but also of the organs and the whole body. The blood according to its nature determined the goodness or badness of the organization; noble blood generated noble men and healthy organs, bad blood a debased posterity and organs predisposed to disease. In scientific works naught remains of these fantastic surmises; they persist like a superstition in lay circles, but no one now maintains their correctness in serious debate. In their stead has arisen the recognition of the particular value of the mother tissue and its cells. These are the factors of inherited properties, the sources of the germs of new tissues and the motor power of vital activity.

During the development of a higher organism the constitution of the individual tissues changes; they become differentiated by means of metaplastic processes which are in their turn connected with cells and cell-territories. Thus it comes about that people have for ages spoken of dissimilar parts. The complete full-grown organism is built up of similar and dissimilar tissues; their harmonious working gives the impression of a unity of the whole organism which is as a matter of fact non-existent. For the further the organism develops the more its social constitution comes into evidence. It consists of innumerable independent parts which together constitute a single social body. If we take the ultimate elements of these parts we must call them all without exception cells, for cells alone are truly alive and scientific judgment is in the last instance concerned with them.

So little is the whole organism a definite unit that the number of its living constituents is in the highest degree inconstant. Looking at the gross structure of organs we are accustomed to regard a certain number of them as typical peculiarities of human beings or the various genera and species of animals. We expect to find two of each paired organ and one of each unpaired in a single individual. Man, like all other mammals, has a fixed number of bones and teeth, and these numbers are rightly used as diagnostic of man or of the particular variety or species of animal. But these numbers form no essential condition of existence; a man with six fingers or seven toes remains a man, just as a lung with supernumerary lobes or a kidney with an excess of coni medullares remains a lung or a kidney. A woman with three, four or even more mammary glands is thereby no more a lower animal than a man with a tail would be. These are theromorphs ("sports") which can have no influence on our opinion as to the sex of the affected individual or its position in the animal scale.

But it will be a long time before general opinion on the significance of "sports" will, even among experts, become unanimous. One sect will connect them with descent, and see in them a proof of atavism; while the other will regard them only as a pathological formation, and will trace this back to an acquired lesion. During the last century we have gone through violent disputes as to whether certain malformations were inherited or acquired. Those who pinned their faith to inheritance had very generally the arriere pensee that the variation was atavistic, and the question soon presented itself as to whether the atavism was derived only from hnman ancestors, or whether one would have to go back as far as the lower animals to account for it. A universally valid explanation of theromorphism has not yet been found. In my opinion it will never be found. Each single example must be separately studied and explained, and the general value of this explanation will be by no means increased if we find atavism in any single case. Doubtless an acquired variation can also be transmitted, and the circumstance that it is animal-like (theroid) does not go to prove a not acquired but atavistically transmitted condition. In connection with this I may refer to my paper on Race Formation and Inheritance.* I can here discuss only the principal ground for the disputes regarding hereditary diseases which are special to pathology.

Medical men are accustomed to describe as hereditary all diseases which reappear in different generations of the same family. Thus one speaks of hereditary arthritis, hereditary

*Published in the "Bastian-Festchrift," Berlin, 1896, pp. 33-38.

tuberculosis, hereditary cancer. It is in fact not difficult to produce genealogical tables which demonstrate the recurrence of a paternal or maternal disease in children or grandchildren. Much trouble has been devoted, in my opinion without result, to seeking the germs of such diseases in the ovum or the semen. One is hence compelled to pass on to generations of cells which took origin after conception. Here we reach what Roux has designated the post-generative formations. The further we pass away from the time of conception the more numerous examples do we find of alterations in the formation of cells and in the formation of embryonic tissues. But there is at the same time the greater possiblity of the alteration having arisen after the formation of the first cells, and hence that the existing cause may have commenced to act at that time. If we set aside this possibility nothing else remains but to assume that from conception, or even from the organs which produced the ovum or the spermatozoon, a predisposition is transmitted which is already present in the earliest cells, even if it can not be recognized in them.

Upon this theory are built up all interpretations of the inheritance of pathological and, we may add, physiological structures. There are, for example, many extraordinary anomalies in the disposition of hair, either through excess or through defect, and nothing is more common than to see the inherited transmission of such anomalies. But hairs are post-generative structures, and a disturbance in their development can make its first appearance only in a latter period of fetal life; not infrequently, indeed, it is first seen after birth. If such a peculiarity recurs through many generations in the branches of a family or a race it is called hereditary, and referred to a hereditary predisposition. But as undoubtedly excesses as well as defects in hairiness are brought about by acquired disturbances, such as actual diseases, it becomes necessary to seek a recognizable cause for such great anomalies as well. If such an one is found, the aid of a predisposition need not, as a rule, be invoked; one may be quite contented with the cause, which is then the causa efficienes.

Very recent medical history affords the most striking example of a rapid and comprehensive change in opinion regarding a disease formerly regarded as hereditary. Leprosy has for thousands of years passed as a contagious disease. But when about a generation ago the number of lepers in Norway increased to an astounding extent, and one family after another was seized with the malady, the question once more came up as to its hereditariness. Zealous investigators ransacked genealogical tables and church-registers, and families were discovered in which leprosy had persisted for decades, or even centuries. So universal was this conviction that the government, with the consent of the clergy, wished to promulgate a marriage-forbidding decree; only a small majority in Parliament threw out the proposition. I was then requested by the government to travel through the leprous districts and to make a report; I succeeded in collecting a certain, though small, number of indubitable cases in which all suspicion of inheritance could be excluded. These were in particular persons who came as healthy adults from quite leprosy-free neighborhoods into the infected districts, and after a long soj urn there developed leprosy.

A few years later Armauer Hansen discovered the leprosy bacillus. Medical opinion changed in a moment. The venerable idea of the contagiousness of the disease was revived. Inheritance was denied and predisposition vanished from the treasure-house of dogmas. I will not assert that the grounds for embracing the present view are absolutely convincing, but I am positive that it is far to be preferred to the dogma of inheritance. And it is an experience instructive to all of us that one single fact, the discovery of a causa viva should have sufficed to dash down the apparently best-grounded theory. The safely established recognition of a known cause has at once converted leprosy from an inherited into an acquired disease.

A similar thing happened a few decades earlier with two skin-diseases which were, according to the views of humoral pathology, traceable to a change in the blood, a dyscrasia, namely, tinea (favus, porrigo) and the itch. The first actually bore the name of tinea hereditaria, or in German erbgrind. But the microscope revealed to Schonlein that favus arises from a mycelial fungus, and as regards scabies the popular Italian view was confirmed, namely, that a mite (Acarus sarcoptes) was its cause. So unstable are the most plausible theories in the light of an objective, practical knowledge.

Exactly the same experience has been met with in relation to certain diseases of the hair. When fungi were found on the hairs, no one cared any more about predisposition, although this possibly does occur. It is certain that there are parasitic forms of alopecia. But fungi can not be found in every case of alopecia. Still less is this the case in anomalies of the hair associated with excessive growth. Here no other explanation is possible, except the assumption of a predisposition. This holds equally for hirsute races and for families of hairy men as well as for those single hairy cutaneous patches (nevus pilosus) which are regarded as hereditary. The factors in the predisposition are the hair-roots, and moreover those which, although arising during fetal life, belong also to the post-generative group, since they are called later into increased activity.

The general cutaneous covering, in brief the "skin," although doubtless a kind of unitary structure of generally similar type, is nevertheless in a double sense a socially constituent organ. Not only is it composed of numberless independent cells and cell-territories of dissimilar kinds; apart from the vessels and nerves, of the connective tissue, the cutis proper, and the horny epithelial tissue, which forms also hairs and glands, the individual constituents of the skin have a special disposition and are exposed to various external and internal influences. This is best shown by the numerous morbid states to the definite scientific classification of which English dermatologists so early devoted themselves. The existence of maculæ, papules, pustules, and all the various other kinds of skin-spots is demonstrated by the fact that in the skin a large number of little communities may be noted from the first as independent or hereditary factors of a particular predisposition. When mothers' marks (nevi), hairs, or even spines grow from them, it follows that in spite of their common origin there must exist a lasting difference between the various localities.

There is another highly remarkable question which every year claims the attention of medical men more and more; this is what was described in the old medicine as aberratio loci, in the new as heterotopia. It has long been known that hairs are present, not alone on the external skin, to which they properly belong, but also in internal organs where they are quite out of place; and further, that other cutaneous structures, such as epidermis, sebaceous glands, and cutis appear in such places. We unite this whole group under the general term "dermoids." Modern histologists have long struggled against this theory of aberration, but they have finally had to quit the field, and the view has become dominant that as a matter of fact even in fetal life smaller or greater rudimentary fragments can be separated from their natural places of abode, and removed to other spots, where they, so to speak, find a new home, and can undergo all the further changes which are dependent on their cutaneous nature. It is thus that cysts and other tumors can arise from them.

The most remarkable examples of such heterotopias are afforded by certain glandular organs which under normal conditions present communities of similar parts, arranged in special divisions. Among them a high place is taken by two organs which have recently demanded much attention, the thyroid and the suprarenal glands. On their surface may often be observed the pushing forward and progressive isolation of separate parts in the form of nodules or small lobes. But occasionally these nodules pass completely out of association with the main body of the gland, and are found disconnected in a perfectly strange place more or less removed from their seat of origin. The farthest journeys are those of the broken-off nodules of the suprarenals; their wanderings lead them to neighboring spots on the kidneys, or even into the interior of those organs, and in other cases over the kidneys into deeper parts of the peritoneum as far as the pelvis. And at all these places they may undergo further change, thus affording starting points for tumor-formation.

The same wandering has long been known in respect of teeth, and one knows that large tumors can arise from misplaced tooth-germs. The like holds with regard to cartilages, in which similar separations are noted in fetal life. The history of rickets has shown that islands of cartilage which were originally connected with the primary cartilages of the epiphyses or diaphyses come later in the course of bone-growth to lie in the interior of the bones, completely separated from their matrix. From them may arise other new formations such as enchondromata and osseous cysts.

Extraordinary, even astonishing, as many of these cases are, they lose the character of perfect strangeness which they exhibit on superficial examination when we recall a frequent heterotopia which was produced at first rather by accident, then in surgical practice, and finally experimentally, and which is known by the name of transplantation. Since the grafting of pieces of the epidermis has come into use in rhinoplasty, and has been applied, often with great success, to the healing of refractory ulcers, it is no longer surprising to think that living pieces of tissue may continue to exist in unwonted situations, and can there undergo further development. Experimentally— and this has also become important in surgical practice—the first place under this head is taken by the transplantation of periosteum, which can be carried into every possible corner of the body, even through the circulation into the lungs, and which in all these places conserves its vitality and also its power of serving as a matrix for osseous tissue.

In my opinion, the bearing of these observations upon medical theory has been overrated, in that a property possessed by the transplanted tissue, to-wit, the property of forming a tumor by proliferation, has been applied to the explanation of tumor-formation in general. This is a mistake. Transplanted tissue has no fresh properties beyond those of the mother tissue from which it is separated.

That a sarcoma can arise from a nevus is only possible because the latter is a part of the skin, and because the skin itself can also produce sarcomata. A cartilaginous tumor can arise from an aberrant piece of cartilage in the middle of a bone, and give rise to an enchondroma, but it may also grow out as a simple cartilaginous hypertrophy (ecchondrosis) from permanent cartilage. A dermoid cyst can serve as the basis for the outgrowth of a cutaneous horn, but cutaneous horns and spines can also grow out of ordinary skin. In each of these cases there is only the realization of a possibility of formation which is present in the matrix in general. At the same time each of these cases illustrates the law of the vita propria of the tissues and of their activity linked to this life.

It is not without great scientific and practical interest to reflect that these observations illustrate another ancient doctrine, the doctrine of parasitism. This doctrine also is traceable back to Paracelsus, who wished to have disease in general regarded as a parasite. One century after another spread this theory abroad, or at least kept its memory green, although there is a fundamental error of logic in assuming the universality of parasitism. For if the living organism is constituted by separate and independent living parts, each of which nourishes itself, and of which most can propagate themselves and perform their special functions, each one of these individual parts must occupy the position of a parasite with respect to the others: it lives on and lessons the common stock of nourishment. The generally accepted view regarding parasitism postulates at the same time the harmfulness of this condition. In reality, every part is endowed with individual life so that it can act prejudicially on the remainder of the organism if its activity becomes excessive or defective. A nevus that becomes a sarcoma can assume a really hurtful significance. Hence it is requisite to remove the sarcoma, but it is not advisable to remove every nevus. Only an access of caution can lead to an operation which finds its sole excuse in the possibility that nevus can conduce to the formation of a sarcoma. In like manner every excessive proliferation (luxuriation) can act harmfully; it may then be described as malignant. But many proliferations are useful, benign or even salutary, as, for instance, the scars which cover a loss of substance. It is just for the sake of a trustworthy prognosis that one must be extremely careful in the application of designations which group whole categories of morbid processes under a common aspect.

The idea of parasitism which we have here discussed in regard to the relation between different parts of the same organism fits in much better where living organisms of a different variety or species enter into an organized corporation, and continue their special life in commensalism. The animal parasites, which exist as entozoa in man and other animals, have been longest known. Since the end of the last century our acquaintance with these entozoa has greatly broadened. Many structures which were formerly regarded as mere bladders (cysts) have been recognized as cestoid worms (entozoa cystical). The trichinæ, apparently sexless animals living in the interior of muscles, were first discovered in this century at Edinburgh; later experimental research succeeded in proving that after the consumption of infected meat these little worms rapidly became sexually ripe in the bowel, and produced not alone ova, but also living embryos and larvæ. Thus one comes to the worms which live in the blood, distomata and filariæ, and which later wander into the tissues. They all have a period during which they have their dwelling as organozoa in the midst of the living tissues of the organism, and become so perfectly incorporated that they carry on their own lives just like the proper cells. Quite new and pertaining exclusively to the investigations of our own time are the parasitic protozoa, beings of so rudimentary a kind that their position in the biological system is even yet not quite clear. Chief among these are the protozoa of malaria, quite microscopical organisms, many of which are such pigmies that they can penetrate into the smallest cells, such as the red blood-corpuscles. The darkness which for thousands of years had enveloped a group of most dangerous diseases—the tropical fevers—has been dispelled by the discovery of these tiny creatures. Important links in the history of these parasites are still wanting; we know nothing definite as to their origin or their career outside the great organisms which are their temporary dwelling-place, and nothing, also, as to their mode of action within these organisms, but we hold the threads by means of which perfect knowledge must be attained.

Lastly comes the equally new field of microscopic plants which appear sometimes as mere grains (cocci), at others as minute rods or chains (bacilli), and from which many of the most severe diseases, the elite of the parasitic infectious maladies, take origin. Their recognition began with the study of two very great and most widely spread processes, fermentation and putrefaction. It will ever remain the imperishable merit of Pasteur, not only to have firmly established the dependence of these processes on the activity of microbes, but to have elucidated the further life history of the germs and their power of producing active chemical or physico-chemical substances. Here for the first time were subjected to experiment parasitic beings which live and carry on their work outside the organism. Hence has been attained the wonderful result which has unlocked new methods both in medicine and in technical science. Above all, the results of microscopical research have been supported by trustworthy experiments, and their significance raised above all doubts; hence pathology in particular has won in directions which had hitherto been shunned by all who studied the nature of its processes, a clearness and certainty which has been reached in few other fields.

The first great stride in the special domain of pathology was made in veterinary medicine. The discovery by Brauell of the anthrax bacillus opened the long series of new, and as we now call them, pathogenic bacilli. It would lead too far afield to refer to all of these, or even to enumerate them; it must suffice to mention the two severest diseases, the dreadful effects of which are accounted for by the action of bacilli—tuberculosis and Asiatic cholera. In both cases it was Robert Koch who was fortunate enough, by means of careful, and in part very delicate procedures, to demonstrate the constant presence of certain bacilli in the organs of patients. At the same time it became plain that in spite of the presence of bacilli in both diseases a totally different kind of infection could be recognized in the two; thus, while tubercle bacilli invade the organs, and therein exhibit their deadly action, the cholera bacillus remains almost exclusively in the intestine, and develops more after the manner of an infusorial plant.

For our discussion to-day it is inadvisable to go into minuter details. Only a few of the greater landmarks can be referred to. One of them I will mention but briefly, as I have written many long papers upon it: the necessity for distinguishing between the cause and the essential nature of infectious diseases. Parasitic beings, including, of course, bacteria, are never more than causes; the nature of the disease depends upon the behavior of the organs or tissues with which the bacteria or their metabolic products meet. From my point of view this distinction is of cardinal importance.

Both my other landmarks require somewhat fuller statement. The first is the general relation of the smaller parasites to the diseases determined by them. Under one name, which reaches back even into the old days of humoral pathology, and which I was the first to introduce into common parlance, are grouped all the processes which are produced by the invasion of morbid substances, under the general designation of infection. The Latin inficere means as one should say to "dirty." The polluting substance (res inficiens) has been called for ages dirt, impuritas. The products of putrefaction (materiæ putridæ) served as its prototype. In Greek they were called miasms (from μιαίνω, inficere), so that these latter names were applied chiefly to such uncleanness as had been produced outside the body. That which had arisen inside the human or animal body was called contagium. Both miasmatic and contagious substances produced by their penetration into the body severe attacks recalling poisoning. To distinguish such a substance from a true poison (venenum) it was designated a virus. The relationship between infection and intoxication was presumed, but it was not without good reason, considering the origin of the impurity, that the difference in designation was retained.

Among the innumerable infectious diseases it was the contagions which, owing to the associated danger to health and life not for individuals only, but for numbers of men and animals, came most prominently to the front. Thus the remarkable property was observed in contagia that they multiplied in the body, and so besides infection as such produced an immeasurable quantity of fresh virulent substance. In this respect they approached living beings, and the thought arose that they themselves were alive (contagia viva). With the discovery of parasitic animals and plants this conjecture soon became a fact. Nothing was easier than to generalize this fact and to assume the presence of independent organisms in each contagious disease. The younger generation of doctors and students disregarded with fiery enthusiasm the necessity of a practical proof, and was filled with the conviction that all infection depended on the invasion of parasitic organisms. And since it was just the severest infections which were produced by the minutest plants and in which bacilli and cocci, or as they are called for short, bacteria, were found in greatest abundance there was circulated for some time that beatific axiom, "Infection is pollution by bacteria."

It was known, however, that parasitic animals and protozoa can also give rise to infection, and that between bacteria and fungi there is more than a slight difference, but for convenience the name of bacteria is retained as a general designation. Further, a peculiar circumstance happened in that for most of the so-called bacteria there were no botanical names. Owing to the novelty of the circumstances in which they are placed, botanists have not even yet succeeded in their customary duty of giving every new plant it special name, of determining its genus and species, and of assigning it to its proper systematic situation. This can easily be understood and forgiven. But it does not in any way alter the erroneousness of a method which attributes every impurity to bacteria on the sole ground of its contagiousness. It may be said that a contagious disease affords suspicions of a bacterial origin, but it should not be called simply bacterial. To do so hinders further research and lulls the conscience to sleep.

Some of the most important contagious diseases have succeeded in resisting the struggle to find in them a parasitic contagium. For example, many have been the sanguine hopes of finding the parasite of venereal disease and as many have been the failures. The coccus of gonorrhea alone has been discovered; the bacterium of syphilis itself remains a pium desiderium. With certainty was it expected that a pathological parasite would be tracked in variola; more than one bacterium was actually found, but none pathogenic. In hydrophobia (lyssa, rabies) all appearances seemed to promise that it would prove to be a microparasitic disease; its contagiousness is undoubted; even a vaccine has, as with smallpox, been prepared, and yet no one has been able to cultivate a specific bacillus. And the same is the case with some of the most dreaded contagious diseases. Painful as it may be one can do nothing but wait, observe, and experiment. Perhaps pathogenic bacteria will be found, but as long as they are not discovered all assumption is useless if not dangerous. To have learnt this is a good omen for a mighty stride in biological methods.

The other, much-further-elaborated point in the study of infectious diseases is the question of the mode of action of infection. As long as infection by animal parasites was regarded as the type of infection in general, the destructive action was described as the result of a mechanical action just comparable to a bite or devouring. But the exact study of the larger entozoa and organoza soon brought about a complete conversion. Neither the Tænia solium nor the Tænia echinococcus possesses an oral opening. They undoubtedly take in nourishment and draw it from their autosites, or, as they are poetically called, their hosts, but this applies only to the absorption of fluids. The feeding of bacteria and other vegetable and plant-like parasites has to be regarded in the same way. They certainly injure the tissues and organs in which they reside by the consumption of important materials, but their action is not limited to this. This much we have already learnt in the study of fermentation and putrefaction. It is admitted that organic matter is destroyed by them, but in addition they produce new substances, some of them eminently poisonous. Thus, it has been known for centuries that alcohol is produced by fermentation. The putrefaction-poisons could for a long time not be isolated; first Selmi and then Brieger achieved this result. Gradually one ptomaine after the other was found; for the whole group the new term of toxins has been introduced by Brieger instead of the name virus. They are in part crystallizable, invariably diffusible, chemical substances which are bound up neither with cells nor other formed elements, though they are produced by the cell-action of the parasites. They are nowadays described by preference as metabolic products, a perfectly platitudinous notion which has far more sound than sense. In former times people were contented to call them secretory products, and I venture to believe that it is better to stand by this term in order not to lose the analogy with glandular secretion.

There are thus two sides to infection; on the one the actual living parasites, on the other their often poisonous secretions. In the individual diseases now one, now the other property comes to the front In the case of the hematobiotic parasites poison-formation may well as a rule count as more important; with those that live in the organs the deprivation of nutriment is much more immediately evident. The invention of artificial nutritive media for bacteria has now provided us with a convenient field for research and observation regarding all these questions.

It would be called carrying coals to Newcastle were I to sketch in London the beneficial effects which the application of methods of cleanliness has exercised upon surgical practice. In the city wherein the man still lives and works who by devising this treatment has introduced the greatest and most beneficent reform that the practical branches of medical science have ever known, every one is aware that Lord Lister, on the strength of his original reasoning, arrived at practical results which the new theory of fermentative and septic process fully confirmed. Before any one had succeeded in demonstrating by exact methods the mircrobes which are active in various diseases, or in establishing the special functions that they perform, Lister had learnt in a truly prophetic revelation the means by which protection against the action of putrefactive organisms can be attained. The opening up of further regions of clinical medicine to the knife of the surgeon and a perfect revolution in the basis of therapeutics have been the consequences. Lord Lister, whom I am proud to be able to greet as an old friend, is already and always will be reckoned amongst the greatest benefactors of the human race. May he be long spared to remain at the head of the movement which he called into existence.

It remains for me to say a word concerning the other great problem, the solution of which the whole world is awaiting with anxious impatience. I refer to the problem of immunity and its practical corollary, artificial immunization. It has already happened once that an Englishman has succeeded in applying this to the nearly complete destruction of at least one of the most deadly infectious diseases. Jenner's noble discovery has stood its trial as successfully, except in the popular fancy, as he hoped. Vaccine is in all hands, vaccination is, with the aid of governments, spreading continually. In the same direction Pasteur worked resolutely, even with boldness; he introduced into practice the vaccines of chicken-cholera, anthrax, and rabies. Others have followed him, and the new doctrine of antitoxins is continually acquiring more adherents But it has not yet emerged from the conflict of opinions. Still less is the secret of immunity itself revealed. Even if everything points to the view that immunity is based on the condition of the cells and the juices of their parenchyma, and not on the serum or the humors—these being probably only the means of transport for the immunizing as for the infecting fluids—we must still become well accustomed to the thought that only the next century can bring perfect light and certainty on those points. The homeopathic notion that toxin and antitoxin are one and the same, seems so foreign to our biological idea that very many experimental and practical proofs will be required before it can be admitted into the creed of the future. Before then we must at least have succeeded in finding a way of strengthening the cells in their fight with bacteria by means of immunity.

Let us, in conclusion, turn once more to the s ecial cells which build up the body, and which arise from proliferation within the body. These exhibit numerous analogies with microbes. They also are independent living beings, or, as Brucke said, elemental organisms implanted in the social structure of the body. They can be removed, transplanted, and grafted in a new situation. If they increase, and thus form a tumor, this can produce metastases by transplantation. But the process as such is always bound up with a certain number of living elements, is always cellular in character. It is not the flowing blood which makes a tumor or a cell; it is the mother cells, from which all new formation originates.

From this consideration I have for decades drawn the conclusion that the local action of the cells, bound up as it is with certain matricial parts, dominates pathological laws, and must also determine the practice of physicians and surgeons. Cellular pathology demands above all treatment attacking the effected parts themselves, be this treatment medical or surgical. It is with great joy that I see this deduction ever becoming more widely generalized, be it with more or with less conscious knowledge. Hence follows in surgery the recommendation of early operation or destruction of the focus of disease.

But cells also, just as bacteria, exercise chemical influences. Apart from the destruction which they effect by absorption, they secrete chemical substances. These appear first as tissue-fluids, passing later into the circulation. Thus arises a change in the composition of the flowing juices, particularly of the blood, in fact a dyscrasia. This is, as I have always explained, a secondary dyscrasia, quite distinct from the primary dyscrasias of the humoral pathologists, by localization of which topical disease, and particularly tumors, were supposed to arise. According to my view, each dyscrasia is determined by the taking in of products of tissue-secretion which may now be called metabolic products, or according to the old dictum, recrementitious substances. The tissue-juice and the excreted material which is returned into the body have of late years gained much esteem. The semen, which I myself have always indicated as the classical example of such a tissue-juice, and exhibited as the prototype of secretion by tumors and organ-cells, has provided materia medica with spermin, as has the thyroid juice with thyroidin and thyroiodin. New substances, some resembling alkaloids, others albuminates, are isolated from various organs, experimentally tested, and technically worked up. So arose injection or serum-therapeutics, on the results of which we are not yet in a position to pass a final judgment, though every one who is sufficiently unprejudiced must admit that they have in many cases been good. Experience will determine the value of these methods; you must learn by the aid of practice to deduce the lasting theoretical truths. But never forget that the source of all these substances and secretions is the cell-activity of living tissue, and that its therapeutical or pathological action on the individual organs or tissues can thus accomplish no aim beyond that of exercising a regulatory influence on cell-activity. May the medical school of Charing Cross Hospital continue upon the newly opened path with zeal and good fortune. But may its students at the same time never forget that neither the physican nor the naturalist dares dispense with a cool head and a calm spirit, with practical observation and critical judgment.—*American Medico-Surgical Bulletin.*

---

De Vlaccos (*Ann. de Gynec. et d'Obstet.*) gives a full report of a distinct instance of precocious puberty, with a photogravure of the patient. She was born in August, 1892, and has all the appearance of a child of ten years, excepting that as far as the genital and mammary regions are concerned she is yet more developed. In height she measures 3 feet 8 inches; her weight is 3 stones 8 pounds. The hair of her head is very abundant, whilst her intellectual development is not above her years. When six months old a bloody discharge was noticed, and it returned in about six weeks. The interval steadily became shorter, and now the catamenia are monthly, lasting for about four days, the child becoming depressed in spirits at each period. The mammæ resemble those of a girl of seventeen years, and not only has hair grown on the pudenda, but much subcutaneous fat has developed in the region of the thighs and nates, as normally occurs at puberty.

